# Arabidopsis TCP Transcription Factors Interact with the SUMO Conjugating Machinery in Nuclear Foci

**DOI:** 10.3389/fpls.2017.02043

**Published:** 2017-11-30

**Authors:** Magdalena J. Mazur, Benjamin J. Spears, André Djajasaputra, Michelle van der Gragt, Georgios Vlachakis, Bas Beerens, Walter Gassmann, Harrold A. van den Burg

**Affiliations:** ^1^Molecular Plant Pathology, Swammerdam Institute for Life Sciences, University of Amsterdam, Amsterdam, Netherlands; ^2^Division of Plant Sciences, C.S. Bond Life Sciences Center and Interdisciplinary Plant Group, University of Missouri, Columbia, SC, United States

**Keywords:** SUMO, TCP, transcription factors, SUMO conjugation, yeast two-hybrid

## Abstract

In Arabidopsis more than 400 proteins have been identified as SUMO targets, both *in vivo* and *in vitro*. Among others, transcription factors (TFs) are common targets for SUMO conjugation. Here we aimed to exhaustively screen for TFs that interact with the SUMO machinery using an arrayed yeast two-hybrid library containing more than 1,100 TFs. We identified 76 interactors that foremost interact with the SUMO conjugation enzyme SCE1 and/or the SUMO E3 ligase SIZ1. These interactors belong to various TF families, which control a wide range of processes in plant development and stress signaling. Amongst these interactors, the TCP family was overrepresented with several TCPs interacting with different proteins of the SUMO conjugation cycle. For a subset of these TCPs we confirmed that the catalytic site of SCE1 is essential for this interaction. In agreement, TCP1, TCP3, TCP8, TCP14, and TCP15 were readily SUMO modified in an *E. coli* sumoylation assay. Strikingly, these TCP-SCE1 interactions were found to redistribute these TCPs into nuclear foci/speckles, suggesting that these TCP foci represent sites for SUMO (conjugation) activity.

## Introduction

Conjugation of the post-translational modification (PTM) Small Ubiquitin-like Modifier (SUMO) is a highly conserved and important mechanism to regulate protein function in eukaryotes. Attachment of SUMO is a dynamic and reversible process affecting foremost nuclear proteins. To this end SUMO is coupled to target proteins by the consecutive action of the SUMO activating (E1 or SAE1) and conjugating (E2 or SCE1) enzymes. In addition, target selection can involve SUMO ligases (E3s). These SUMO E3s are, however, not essential for the modification of each substrate. The genome of Arabidopsis (*Arabidopsis thaliana*) encodes eight SUMO paralogs of which only four are expressed (Kurepa et al., 2003; Lois et al., 2003; Novatchkova et al., 2004). Of these four genes, *SUMO1* and *SUMO2* embody the “archetype” SUMO genes and they are highly related (sharing 89% identity at the protein level) (Hammoudi et al., 2016). These two Arabidopsis genes act redundantly and combined they are essential (Saracco et al., 2007). In contrast, the other two genes, *SUMO3* and *SUMO5*, have likely diversified in terms of their function and the biochemical activity of the gene product (van den Burg et al., 2010; Castaño-Miquel et al., 2013; Hammoudi et al., 2016). SCE1, which is encoded by a single gene in Arabidopsis, catalyzes conjugation of SUMO to substrates by forming an isopeptide bond between the C-terminus of mature SUMO and a lysine side chain in substrates. Specific residues in the catalytic site of SCE1 recognize and interact with a short peptide motif found in substrates, ΨKxE, which includes the acceptor lysine—hereafter called the SUMO acceptor site (SAS) (Bernier-Villamor et al., 2002; Yunus and Lima, 2009; Matic et al., 2010). In this motif, Ψ denotes a hydrophobic residue, while x denotes any residue.

The best-characterized Arabidopsis SUMO E3 ligase is SIZ1 (SAP and Miz 1) (Miura et al., 2005). SIZ1 was shown to stimulate SUMO attachment to several targets including SCE1, but also to transcription regulators like GTE3 (GLOBAL TRANSCRIPTION FACTOR GROUP E3), ICE1 (INDUCER OF CBP EXPRESSION 1), and ABI5 (ABSCISIC ACID (ABA)-INSENSITIVE 5) (Garcia-Dominguez et al., 2008; Miura et al., 2009; Miura and Ohta, 2010). Other studies have shown that SIZ1 controls sumoylation of the ubiquitin E3 ligase COP1 (CONSTITUTIVE PHOTOMORPHOGENIC 1) and the protein kinase SnRK1 (SNF1-RELATED KINASE 1) (Crozet et al., 2016; Lin et al., 2016). For each of these targets a direct interaction with SIZ1 could be shown, indicating that SIZ1 plays a role in their recognition. The SUMO isoforms accumulate as precursors, which need to be maturated by SUMO proteases to expose their conserved C-terminal di-glycine motif. The same SUMO proteases also remove SUMO from modified proteins. Certain SUMO proteases display substrate specificity, which was linked to their (sub)cellular localization (reviewed by Hickey et al., 2012). SUMO can also bind non-covalently to other proteins when they contain a SUMO-interacting motif (SIM) (Hecker et al., 2006). In several cases, SUMO conjugation at non-consensus sites appeared to depend on SIMs that were present in these substrates (Lin et al., 2006; Saleh et al., 2015). To date, all SIMs consist of a hydrophobic core sequence (containing Leu, Val, and/or Ile residues), which can be preceded or succeeded by an acidic region consisting of Glu, Asp, and/or phosphorylated Ser and Thr residues (Hecker et al., 2006; Kerscher, 2007).

Hundreds of SUMO substrates have now been identified in different model systems, including Baker's yeast, human cell lines, and Arabidopsis using proteomics (Denison et al., 2005; Vertegaal et al., 2006; Wohlschlegel et al., 2006; Golebiowski et al., 2009; Miller et al., 2010, 2013; Park et al., 2011). These proteomics studies have exposed that more than 400 different proteins can be sumoylated in Arabidopsis (Miller et al., 2010, 2013). These studies also revealed that at least 85 Arabidopsis transcription factors (TFs) are substrates for sumoylation. However, purification of SUMO modified proteins from cell lysates remains challenging, as their levels are low due to the high SUMO protease activity in these lysates. Another method to identify putative SUMO substrates is the yeast two-hybrid (Y2H) assay, which has been successfully used to identify more than 200 putative Arabidopsis sumoylation targets and/or interactors of the SUMO (de)conjugation pathway (Elrouby and Coupland, 2010; Elrouby et al., 2013). In these two studies four bait constructs were used to screen a random library, namely two SUMO paralogs (SUMO1 and SUMO3), SCE1, and the SUMO protease ESD4 (EARLY IN SHORT DAYS 4). A substantial fraction of the identified interactors and/or substrates were nuclear proteins that participate in a wide range of processes including RNA biogenesis, chromatin remodeling, DNA maintenance and, importantly, transcriptional regulation (Elrouby and Coupland, 2010; Miller et al., 2010, 2013; Mazur and van den Burg, 2012; Elrouby et al., 2013).

As the expression of TFs is tightly regulated and highly depends on the cell type, the developmental stage, and/or the presence of external stimuli, it is difficult to exhaustively identify protein-protein interactions for low abundant TFs using random Y2H libraries as TFs are underrepresented in such libraries. We here used a collection of Arabidopsis TFs (the REGIA collection) consisting of more than 1,100 clones (Paz-Ares, 2002). This collection consists of full-length open reading frames (ORFs) that are cloned in a yeast two-hybrid prey vector, which was then introduced into yeast (GAL4-AD; To et al., 2012). Previously this arrayed library was successfully used to screen for interactors of TOPLESS (TPL) and TPL-related (TPR) co-repressors (Causier et al., 2012).

To systematically extend the current list of TFs that can potentially serve as SUMO substrates, we screened this REGIA collection using as baits three SUMO paralogs (SUMO1,−2,−3), SCE1, SIZ1, and two SUMO proteases, ESD4 and OTS2 [OVERLY TOLERANT TO SALT 2, also known as ULP1c (Ubiquitin-like-specific protease 1C)]. We identified 76 TFs as putative interactors of the SUMO (de)conjugation pathway. Among others, a large number of TCPs (TEOSINTE BRANCHED1/CYCLOIDEA/PROLIFERATING CELL FACTOR 1) was identified. The TCPs represent a plant-specific family of TFs, which is implicated in plant development, hormone signaling, and plant immunity (Guo et al., 2010; Tao et al., 2013; Davière et al., 2014; Kim et al., 2014; Sugio et al., 2014). Using Bimolecular fluorescence complementation (BiFC), we confirmed the interaction of these TCPs and found that the interaction with SCE1 redirects certain TCPs to nuclear speckles/bodies. These TCPs interacted directly with SCE1 but not with a catalytic-dead variant of SCE1, suggesting that they are direct SCE1 substrates. In agreement, these TCPs were readily sumoylated in *E. coli* using a reconstituted SUMO conjugation assay (Okada et al., 2009). Together, this study thus indicates that sumoylation of TCPs potentially affects their function and (sub)nuclear localization in Arabidopsis, which might affect their reported roles in plant development and/or immunity.

## Results

### Identification of arabidopsis TFs as sumo substrates/interactors using Y2H

We performed a Y2H screen to exhaustively screen for TFs that interact with the SUMO machinery. As baits, we used two SUMO variants: mature SUMO (GG) and a conjugation-deficient variant of SUMO (that lacks it's C-terminal diGly motif needed for isopeptide bond formation, hereafter ΔGG). With these two variants Y2H interactions were tested for three different paralogs (Arabidopsis SUMO1,−2, and −3). This allowed us to distinguish between interactors (ΔGG) and potential SUMO conjugation substrates (GG). We also used as baits the SUMO E2 enzyme SCE1 and the E3 ligase SIZ1, as both proteins are implicated in substrate selection. We also screened with two different SUMO proteases, ESD4 and ULP1C (UBIQUITIN-LIKE-SPECIFIC PROTEASE 1C; a.k.a. OVERLY TOLERANT TO SALT 2), as SUMO proteases are also known to provide substrate specificity. Fusion of the GAL4 DNA binding domain (BD) to the SUMO isoforms and to the wild type variant of SCE1 did not cause auto-activation of the *GAL4* promoter in yeast (Figure S1). In contrast, fusion of the GAL4 BD to the full-length SIZ1 protein resulted in auto-activation of the *GAL4* promoter (Figure S1), as seen by others (Garcia-Dominguez et al., 2008). For this reason we only used the N-terminal half of SIZ1 with the PINIT region (residues 1–536) in our Y2H screen, as previously used by others (Garcia-Dominguez et al., 2008). Structural studies have shown the importance of the PINIT domain for binding of the yeast substrate PCNA to yeast SIZ1 (Streich and Lima, 2016). In the case of ESD4, it was reported that the full-length protein did not accumulate to detectable levels in yeast (Elrouby and Coupland, 2010). However, we were able to express a full length BD-fusion of both ULP1C and ESD4 (Figure S2). To obtain comparable data we also screened with a catalytically-dead variant of ESD4 and ULP1C (C448S and C512S, respectively) and with a fragment of these two proteases in which the N-terminal half containing the regulatory domain was fused to the GAL4 BD (1–285 and 1–345, respectively). Both these variants were previously used for ESD4 (Elrouby and Coupland, 2010). The truncated form of ULP1C (1–345) was, however, excluded from our studies, as it caused auto-activation of the *GAL4* promoter (Figure S1).

**Figure 1:**
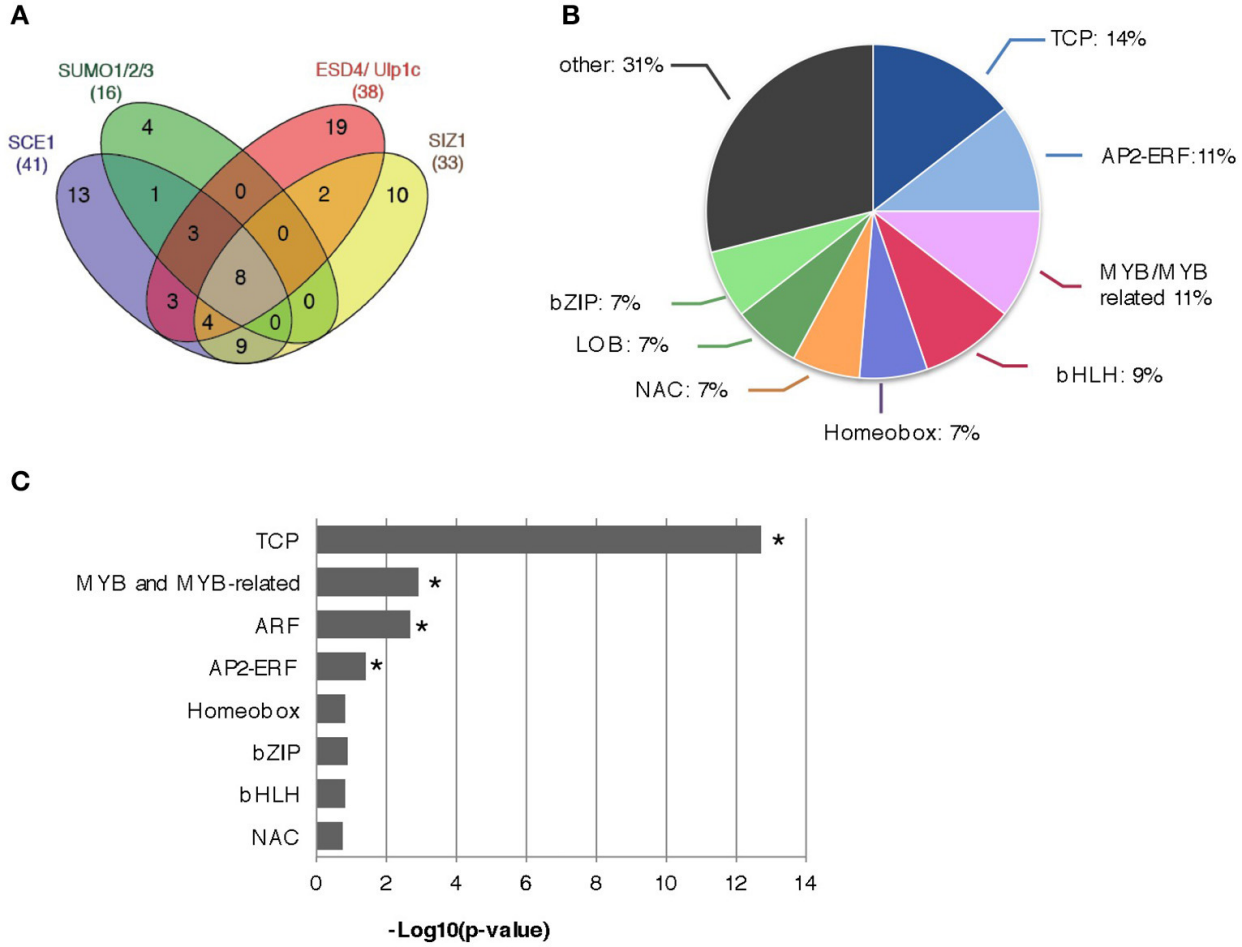
Summary of the Y2H protein-protein interactions found between the Arabidopsis REGIA TF collection and the proteins that control SUMO (de)conjugation. **(A)** Venn diagram showing the number of overlapping interactions between SCE1, SUMO1/2/3, ESD4/ULP1C, and SIZ1. The numbers in brackets indicate the total number of interactors found per bait (set). **(B)** Pie diagram representing different Arabidopsis TF families (min. 5 members of single TF family) that were most often found to interact with proteins of the SUMO (de)conjugation pathway. **(C)** The TF families TCP, MYB, and MYB-related, ARF, and AP2-ERF are significantly enriched amongst the set of interactors. Binomial test; ^*^significant enrichment (*p* < 0.05).

In total we determined for 13 different bait constructs whether they interacted with any TF in the REGIA Y2H library, resulting in 15,184 different protein-protein interaction tests. The screens were conducted in technical triplicate and only when yeast growth was seen in at least two replicates we scored the interaction as positive (Table 1). Overall we found 41 interactors for SCE1, 33 for SIZ1(1–536), a combined number of 39 interactors for the two SUMO proteases (i.e., for the five tested variants of ESD4 and ULP1C) and 16 interactors for the three SUMO paralogs combined (Figure 1A). The interactions found for the mature SUMO proteins (GG) overlapped strongly with those found for the conjugation-deficient variants (ΔGG), especially for SUMO3 (100% overlap; Table 1) indicating that these interactions with SUMO3 potentially represent direct protein-protein interactions rather than conjugation of SUMO3 to these proteins by the yeast SUMO E2 enzyme (Ubc9). However, SCE1 and SUMO3 interacted both with the same eight TCPs and seven of these eight TCPs interacted also with SUMO1(ΔGG), but not SUMO2(ΔGG) despite the high homology between SUMO1 and −2. We identified eight proteins that interacted with SCE1, SIZ1(1–536) and at least one variant of the SUMO proteases and one variant of the SUMO paralogs (Figure 1A). Combined, we found more than 15 distinct TF families in these screens (Table 1 and Figure 1B) (based on the classification of the Arabidopsis TF by DATF, http://datf.cbi.pku.edu.cn) including the TCP, bHLH, AP2/ERF, MYB+MYB-related, bZIP, homeobox, and NAC families (Table 1 and Figure 1B). In particular, the TCP, MYB+MYB-related, ARF, and AP2/ERF families were found to be significantly overrepresented in our list of interactors (Figure 1C).

**Table 1:** List of SUMO interactors identified in the Y2H screens.

**AGI code**	**Name**	**Family**	**SCE1**	**SIZ1 1–536**	**ESD4**	**ESD4 1-285**	**ESD4 C448S**	**Ulp1C**	**Ulp1C C512S**	**SUMO1GG**	**SUMO1ΔGG**	**SUMO2GG**	**SUMO2ΔGG**	**SUMO3GG**	**SUMO3ΔGG**
AT3G45150	TCP16	TCP	3	3	y	3	y	y	3	y	y	y	y	y	y
AT3G15030	TCP4	TCP	3	3	y	3	3	y	3	y	y			y	y
AT3G47620	TCP14	TCP	3	3	y	3	y	y	3	y	y			y	y
AT1G53230	TCP3	TCP	3	y	3	3	y	y		y	y	y		y	y
AT1G35560	TCP23	TCP	3	y	y		y	y	3	y	y			y	y
AT1G67260	TCP1	TCP	y	y	y	3	y	y	3		y			y	y
AT1G69690	TCP15	TCP	3	3	y	3	y	y	3		y			y	y
AT5G51910	TCP19	TCP	3		y	3			3					y	y
AT5G23260	AGAMOUS-like32	MADS	3				y		y	y					
AT4G36710	HAM4	GRAS	3	3		y			3	y					
AT1G26960	HB23	Homeobox	3	3			3								
AT3G58190	LBD29	LOB	y	3					y						
AT4G18390	TCP2	TCP	y	y					3						
AT4G36060	bHLH11	bHLH	3	3			y								
AT2G16770	bZIP23	bZIP	y			y				y					
AT4G14410	bHLH104	bHLH				3	y		y						
AT5G04150	bHLH101	bHLH				3	y		y						
AT4G36990	HSFB1	HSF				3	y		y						
AT1G66230	MYB20	MYB	y	3											
AT1G72830	HAP2C	CCAAT-HAP2	y	3											
AT3G06120	MUTE	bHLH	y	3											
AT3G17609	homolog of HY5	bZIP	y	y											
AT5G11260	HY5	bZIP	y	3											
AT3G61830	ARF18	ARF	y	3											
AT5G03510	C2H2-type	C2H2	y	3											
AT5G06080	LBD33	LOB	3	3											
AT5G45980	WOX8	Homeobox	y	y											
AT1G58100	TCP8	TCP	y		y										
AT5G05410	DREB2A	AP2-ERF	3				y								
AT3G11020	DREB2B	AP2-ERF	3									y			
AT1G14200	RING/U-box	C3H	y			y									
AT1G18330	EPR1	MYB					3		y						
AT1G32510	NAC011	NAC		3					y						
AT3G01470	HB-1	Homeobox		3	3										
AT3G04420	NAC048	NAC				y	3								
AT5G06950	AHBP-1B	bHLH				3	y								
AT5G15060	LBD	LOB				y			y						
AT5G61820	AT5G61820	unclassified				y			y						
AT1G51140	BHLH3	bHLH												3	3
AT3G61630	CRF6	AP2-ERF												y	3
AT1G53160	FTM6	SPL												y	y
AT1G22190	ABR1	AP2-ERF	3												
AT4G17490	ERF6	AP2-ERF	y												
AT1G04370	ERF14	AP2-ERF	3												
AT1G63100	GRAS	GRAS	y												
AT1G72210	bHLH96	bHLH	3												
AT3G13445	TBP1	unclassified	y												
AT3G23250	MYB15	MYB	y												
AT5G10280	MYB64	MYB	y												
AT5G41410	BEL1	Homeobox	y												
AT2G45650	AGAMOUS-like 6	Homeobox	y												
AT3G02150	TCP13	TCP	y												
AT2G33860	ARF3	ARF	3												
AT1G55600	WRKY	WRKY	y												
AT1G56170	HAP5B	CCAAT-HAP5		3											
AT2G40220	ABI4	AP2-ERF		3											
AT1G59530	BZIP4	bZIP		3											
AT2G22750	bHLH18	bHLH		3											
AT1G18780	RING/U-box	C3HC4		y											
AT5G18300	ANAC088	NAC		3											
AT5G37415	AGL105	MADS		y											
AT3G09600	LCL5,RVE8	MYB		3											
AT3G12720	MYB67	MYB		y											
AT4G35550	WOX13	Homeobox		3											
AT5G15840	B-BOX1	C2C2-CO-like				y									
AT3G15270	SPL5	SPL				y									
AT1G32870	NAC13	NAC				y									
AT1G54330	NAC020	NAC				y									
AT5G56500	CPN60beta3	unclassified				3									
AT5G66870	LBD36	LOB				3									
AT2G44410	STUbL6	STUbL^*^					y								
AT3G11090	LBD21	LOB							y						
AT3G27920	MYB0	MYB							y						
AT1G26780	MYB117	MYB							y						
AT5G52830	WRKY27	WRKY							y						
AT5G47230	ATERF-5	AP2-ERF													3
		Total:	41	33	10	23	18	7	22	8	7	3	1	11	12

The interactions between SUMO proteins and SUMO (de)conjugation enzymes and the preys are indicated with gray boxes; dark gray represent yeast growth on selective medium (−WLH) supplemented with 1 mM 3-AT, while light gray indicates yeast growth on selective medium without 3-AT supplementation. Each interaction was tested in triplicate and the interaction was scored positive when yeast growth was seen for at least two replicates.

* *Not a true TF but present in the REGIA collection*.

### Interactors of SCE1 contain both SUMO attachment sites and SIMs

**Figure 2:**
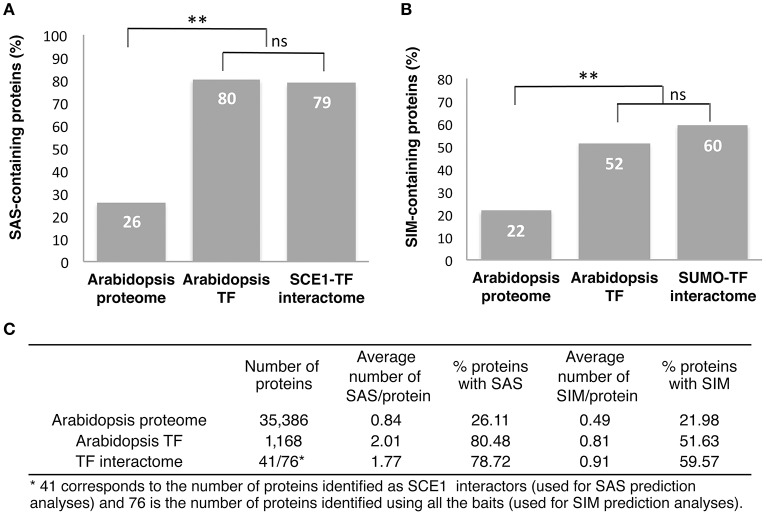
Most of the REGIA TFs that interacted with SCE1 contain a predicted SUMO acceptor site and/or SUMO interaction motif. **(A)** Percentage of proteins that contain at least one putative SUMO acceptor site (SAS) in the Arabidopsis proteome, all Arabidopsis TFs and the TFs that interact with SCE1 (SCE1-TF interactome). **(B)** Percentage of proteins containing at least one putative SUMO interaction motif for the Arabidopsis proteome, all Arabidopsis TFs and SCE1-TF interactome. In both **(A,B)** a binomial test was used; ^**^ significant enrichment (*p* < 0.01), while ns means not significant. **(C)** Table showing the overall number of proteins analyzed in **(A,B)**, the average number of SAS and SIM motifs per protein, and the percentage of proteins with these two motifs. Both motifs were predicted using GPS-SUMO.

A commonly used tool to predict SAS and SIM motifs is GPS-SUMO (Ren et al., 2009; Zhao et al., 2014). We used it to predict these motifs in our set of SCE1 interactors. Of the proteins that interacted with SCE1, 79% contained at least one SAS, with an average of 1.77 motifs per protein (Figures 2A,C). As reference, we also predicted SAS for all the protein models in the Arabidopsis genome (TAIR10). Twenty-six percent of these protein models contained at least one SAS, with an average of 0.84 motifs per protein model (Figures 2A,C). This enrichment of SAS motifs in our set of Y2H interactors is comparable to that found by others (Golebiowski et al., 2009; Miller et al., 2010). However, TFs themselves already show a significant increase in the number of predicted SAS motifs, i.e., 80% of them contained at least one SAS motif with an average of 2.01 motifs per protein model, which supports the general notion that TFs often act as substrates for sumoylation.

Likewise, 60% of our SUMO interactors contained at least one putative SIM, with an average of 0.91 motifs per protein (Figures 2B,C). As a reference we used again the entire set of predicted Arabidopsis protein models and found that only 22% of these models was predicted to contain at least one SIM (with an average of 0.49 motifs per protein). Thus, we find an increase in the percentage of SIMs in our list of interactors, but this is hardly more than the background, i.e., we found that 52% of Arabidopsis TFs contained at least one SIM. Hence, we find that the TFs in general contain an increased number of SAS or SIM motifs and that our Y2H screen did not further (strongly) enrich for proteins with these motifs.

### TCPs interact specifically with SCE1 through its catalytic site

**Figure 3:**
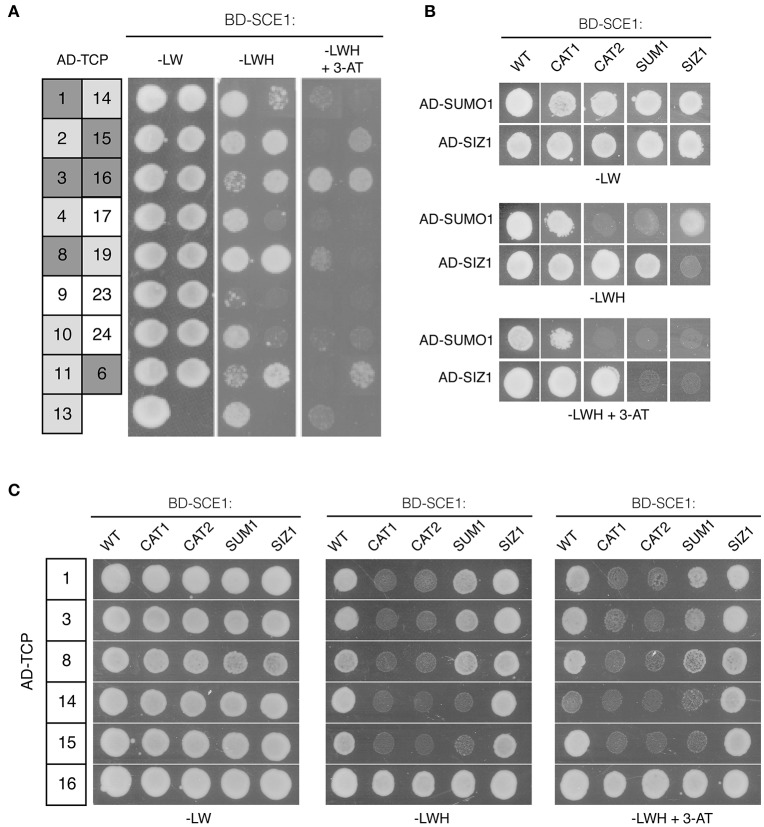
An intact catalytic pocket of SCE1 is essential for its interaction with the different TCPs. **(A)** Y2H interaction assay between wild type SCE1 (BD-SCE1) and different Arabidopsis TCPs (AD-TCP; the number denotes the TCP TF). Gray boxes reflect a positive protein-protein interaction with dark gray depicting yeast growth on selective medium (–LWH) supplemented with 1 mM 3-AT (strong interaction), while light gray depicts yeast growth only on −LWH (weak interaction). **(B)** Y2H assay showing the interaction between different SCE1 variants and SUMO1 or SIZ1. SCE1 variants: CAT1, catalytically dead-variant of SCE1(C94S); CAT2, catalytically dead-variant of SCE1 in which the ΨKxE binding pocket is mutated (Y87A C94S C94S D129A); SUM1, SCE1 variant in which the non-covalent binding site of SUMO is mutated (R14E R18E H20D); SIZ1, SCE1 variant with mutations in the SIZ1 binding site (P70A P106A). None of these SCE1 variants showed auto-activation (Figure S1). **(C)** Six TCPs, which strongly interacted with SCE1 **(A)**, were tested for their interaction with SCE1. Except for TCP16 all of the tested TCPs failed to interact with SCE1^CAT1^ and SCE1^CAT2^. Both TCP14 and TCP15 fail to interact with SCE1^SUMO1^. –LW, minimal medium lacking Leu/Trp; –LWH, medium lacking Leu/Trp/His (weak interaction); + 3-AT (1 mM), inhibitor for low-level constitutive expression of the *HIS3* reporter gene. Yeast growth was scored after 3 days at 30°C and the experiment was performed three times with similar results.

Our screen yielded several TCPs that interacted with multiple baits. Other groups also identified many TCPs as interactors in their Y2H screens with unrelated baits, suggesting that these TCPs readily interact in the Y2H. For example, TCP8, TCP14, and TCP15 were found to interact with various effector proteins of unrelated plant pathogens (Sugio et al., 2011, 2014; Weßling et al., 2014; Janik et al., 2017; Yang et al., 2017), but these TCPs also interacted with SUPPRESSOR OF rps4-RLD1 (SRFR1), a negative regulator of effector-triggered immunity (Kim et al., 2014). In this latter case, these interactions were only found with one class of TCPs suggesting that they were *bona fide* interactions. To further delineate the specificity of our interactions, we isolated 17 of the TCP clones in the REGIA library. Instead of using yeast mating, we now co-transformed each clone together with SCE1 into yeast to re-confirm the interaction with SCE1 (Figure 3A). Except for TCP17,−23, and −24, all the tested TCPs interacted with SCE1. For TCP1,−3,−6,−8,−14,−15, and −16 the interaction remained even when 1 mM 3-amino-1,2,4-triazole (3-AT) was added to the plates (Figure 3A).

In order to establish the specificity of the TCP-SCE1 interactions, we tested whether six of these TCPs (the strongest interactors of SCE1) can still interact with SCE1 when it is functionally mutated. To this end, we identified conserved residues in Arabidopsis SCE1 that are important for the non-covalent interaction of SCE1 with SUMO1 and/or SIZ1. Studies on the human and yeast SUMO E2 proteins had shown that the residues Arg14, Arg18, and His20 of SCE1 are likely important for the non-covalent interaction between SCE1 and SUMO (Bencsath et al., 2002). Likewise, it was shown that two prolines (Pro70 and Pro106 in SCE1) are critical for the interaction between the human SUMO E2 enzyme (HsUbc9) and the PIAS family of SUMO E3s (Mascle et al., 2013). This latter protein family is closely related to Arabidopsis SIZ1. As these five residues are strictly conserved in Arabidopsis SCE1, we mutated them (i.e., for Arg14Glu Arg18Glu His20Asp in SCE1^SUM1^ and Pro70Ala and Pro106Ala in SCE1^SIZ1^) to examine if the TCP-SCE1 interactions are direct or indirect, i.e., whether they depend on the formation of a trimeric protein complex that includes the yeast SUMO protein (Smt3) or the yeast SIZ1 protein. We also mutated the catalytic site of SCE1 (Cys94Ser in SCE1^CAT1^), which prevents the formation of an active SCE1~SUMO1 thioester complex (Reverter and Lima, 2005). Structural studies had also demonstrated that the SUMO acceptor motif (ΨKxE) is directly recognized by the E2 enzyme. Based on these structural studies with the human and yeast SUMO E2s, we decided to also mutate Tyr87, Ser89, and Asp129 (SCE1^CAT2^), as these three residues coordinate the binding of the ΨKxE peptide (Bernier-Villamor et al., 2002; Reverter and Lima, 2005; Mohideen et al., 2009).

Using the Y2H assay, we tested if SUMO1 and SIZ1 indeed did not interact with SCE1^SUM1^ and SCE1^SIZ1^. First, we examined if the variants accumulated in yeast (Figure S2). To our surprise, both the wild type SCE1 and SCE1^CAT1^ accumulated to lower levels in yeast than SCE1^CAT2^, SCE1^SUM1^, and SCE1^SIZ1^. Apparently, these latter substitutions have a stabilizing effect on the BD-SCE1 fusion protein in yeast. In agreement with studies on the yeast Ubc9 protein (Mascle et al., 2013), Arabidopsis SCE1^SUM1^ could not interact with SUMO1 in yeast (Figure 3B). Unexpectedly, SCE1^CAT2^ also failed to interact with SUMO1, while SCE1^CAT1^ appears to interact with SUMO1 like wild-type SCE1 (Figure 3B). As predicted, the SCE1^CAT1^ and SCE1^CAT2^ mutants still interacted with SIZ1, suggesting that the catalytic dead variants can still adopt a native protein fold (Figure 3B). In agreement with previous reports, no interaction was observed between the full length SIZ1 AD-fusion protein and the BD-SCE1^SIZ1^ fusion (Figure 3B), indicating that the two conserved prolines of SCE1 (Pro70 and Pro106) are crucial for this interaction with SIZ1. Importantly, SCE1^SIZ1^ also showed weaker interaction with SUMO1 (Figure 3B), suggesting that this SCE1·SUMO1 interaction is stabilized by binding of a yeast SUMO E3.

We then tested if the TCP proteins could still interact with these SCE1 mutants. Except for TCP16, all the tested TCPs failed to interact with both SCE1^CAT1^ and SCE1^CAT2^ (Figure 3C), suggesting that they directly bind to the catalytic pocket of SCE1 and as such they are likely direct substrates of SCE1 (i.e., their sumoylation would be independent of SIZ1). Moreover, TCP14 and −15 failed to interact with SCE1^SUM1^ (Figure 3C). This latter result suggests that the non-covalent binding of SUMO to a TCP14/15·SCE1 complex might stabilize these interactions.

### BiFC confirms that the TCP transcription factors interact with SCE1 *in Planta*

**Figure 4:**
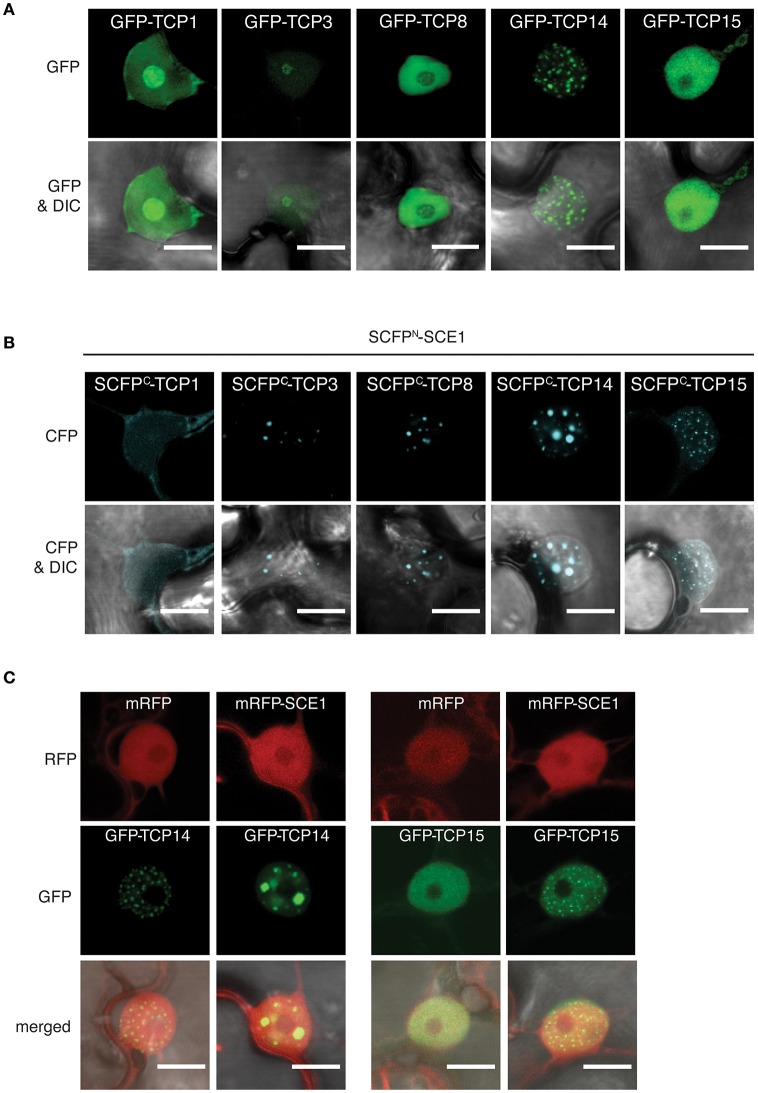
The TCP proteins relocalize to nuclear bodies or punctate structures in the nucleus when bound to SCE1. **(A)** Nuclear localization of the GFP-TCP1,−3,−8,−14, and −15 fusion constructs upon transient expression in *N. benthamiana*. **(B)** Nuclear localization of the BiFC complex formed between SCFP^N^-SCE1 and SCFP^C^-TCP1, −3, -TCP8, -TCP14, and -TCP15. The speckles for the SCE1-TCP14 BiFC pair are enlarged compared to GFP-TCP14. **(C)** Co-expression of mRFP-tagged SCE1 changes the localization of GFP-TCP14 and GFP-TCP15. All fusion constructs were transiently (co-)expressed in *N. benthamiana* epidermal leaf cells using *Agrobacterium*. Scale bar = 10 μm. The fluorescence filters are indicated on the left; DIC, differential interference contrast microscopy.

In order to verify the TCP-SCE1 interactions, we performed bi-fluorescence complementation (BiFC) assays. We only tested TCP1,−3,−8,−14, and −15, as they specifically interacted with SCE1 in our Y2H assay. TCP8,−14, and −15 belong to the Class I TCPs, while TCP1 and −3 belong to the Class II TCPs (Martín-Trillo and Cubas, 2010). First, we examined the subcellular localization of these five TCPs alone by transiently expressing them as GFP-tagged proteins in *N. benthamiana* using *A. tumefaciens*. Each of the five GFP-TCP proteins localized exclusively to the nucleus (Figure 4A). However, GFP-TCP1 and GFP-TCP3 localized to both the nucleoplasm and the nucleolus, while GFP-TCP8 and GFP-TCP15 showed only a diffuse signal in the nucleoplasm. Strikingly, GFP-TCP14 localized exclusively in nuclear foci. However, in other studies TCP8 and TCP14 were shown to localize to nuclear foci (Mukhtar et al., 2011; Valsecchi et al., 2013; Kim et al., 2014; Yang et al., 2017).

Next, we verified the interactions between SCE1 and the five TCPs *in planta* using BiFC. To this end, the N-terminal half [1–173] of Super Cyan Fluorescent Protein (SCFP) was fused to the N-terminus of SCE1 (SCFP^N^-SCE1), while the C-terminal half [156–239] of SCFP was fused to the N-terminus of the TCPs (SCFP^C^-TCP) (Gehl et al., 2009). SCE1 interacted with each of these TCPs in the nucleus although the signal was faint for TCP1 (Figure 4B). Noticeably, the reconstituted signal for CFP was foremost found in nuclear foci for the BiFC pairs of TCP3,−8,−14, and −15 with SCE1. These nuclear foci differed in size and number for these three TCPs. For example, the TCP14·SCE1 BiFC pair localized in a small number of nuclear foci, which were increased in size in comparison to GFP-TCP14 (Figures 4A,B). As negative control, we co-expressed the SCFP^C^-TCP fusions with SCFP^N^-GUS to correct for background fluorescence reconstitution. We did not find any CFP signal in the nucleus when we co-expressed the SCFP^C^-TCP fusions together with SCFP^N^-GUS (Figure S3). Apparently, the localization of the TCPs is affected by their interaction with SCE1. To confirm this, we overexpressed GFP-TCP14 or -TCP15 together with SCE1 fused to the monomeric variant of Red Fluorescent Protein (mRFP-SCE1) or with mRFP alone (negative control) to see if overexpression of SCE1 is sufficient to relocalize TCP14 or−15 to subnuclear complexes (Figure 4C). As expected, we found nucleoplasmic localization for SCE1, but the localization pattern of TCP14 and TCP15 changed upon co-expression of mRFP-SCE1. Both TCP14 and TCP15 localized in this latter case to nuclear foci that resembled those seen for the SCE1·TCP14 and SCE1·15 BiFC pairs. Combined, the Y2H and microscopy data suggest that these TCP family members interact specifically with SCE1 and that this interaction results in their accumulation in subnuclear foci.

### TCP proteins are modified in the reconstituted sumoylation assay in *E. coli*

**Figure 5:**
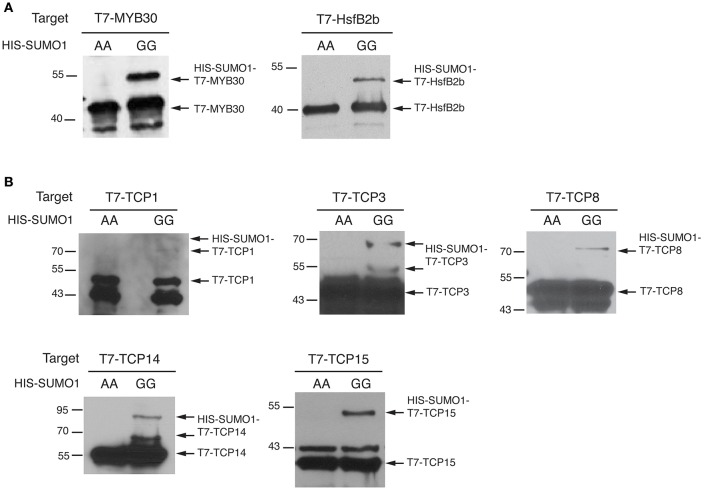
TCP transcription factors are sumoylated in a reconstituted sumoylation assay in *E. coli*. **(A)** *E. coli* sumoylation assay with MYB30 and HsfB2b, two known SUMO conjugation substrates. Both proteins were fused to the T7-tag; only when HIS-SUMO1^GG^ and not HIS-SUMO1^AA^ is co-expressed are both proteins SUMO-modified in *E. coli*. The substrates were detected using an antibody against the T7-tag. The molecular weights (kDa) for the protein standards are indicated on the left. **(B)** Similar to **(A)** except that the sumoylation of TCP1,−3,−8,−14, and -TCP15 is tested. Original blots provided in Supplementary Presentation 1.

To confirm that these five TCPs can serve as a direct SUMO substrate for the SCE1 enzyme, we tested their sumoylation in a reconstituted sumoylation assay in *E. coli* (Okada et al., 2009). In this system the *A. thaliana* SUMO E1 and E2 enzymes are co-expressed together with SUMO and a putative substrate. As positive control, we used two known SUMO substrates from Arabidopsis, i.e., the transcription factors MYB30 (Okada et al., 2009) and HsfB2b (HEAT STRESS TRANSCRIPTION FACTOR B-2b) (Miller et al., 2010). SUMO conjugation is seen by the appearance of high-molecular weight forms of the tested proteins. Using immunoblot analysis we confirmed that both MYB30 and HsfB2b are readily sumoylated by the mature SUMO1 (SUMO1^GG^), but not when a variant of SUMO1 is expressed in which the diglycine motif is replaced by two alanines (SUMO1^AA^) (Figure 5A; original WBs provided as Figure S3). In the same way, we tested the sumoylation of the five TCPs and found that they all were readily sumoylated in *E. coli* (Figure 5B). For TCP8 and TCP14, a double band was found, which could signify poly/multi-sumoylation (chain formation or modification of multiple lysines, respectively).

### The TCP domain is not sufficient for the interaction with SCE1

**Figure 6:**
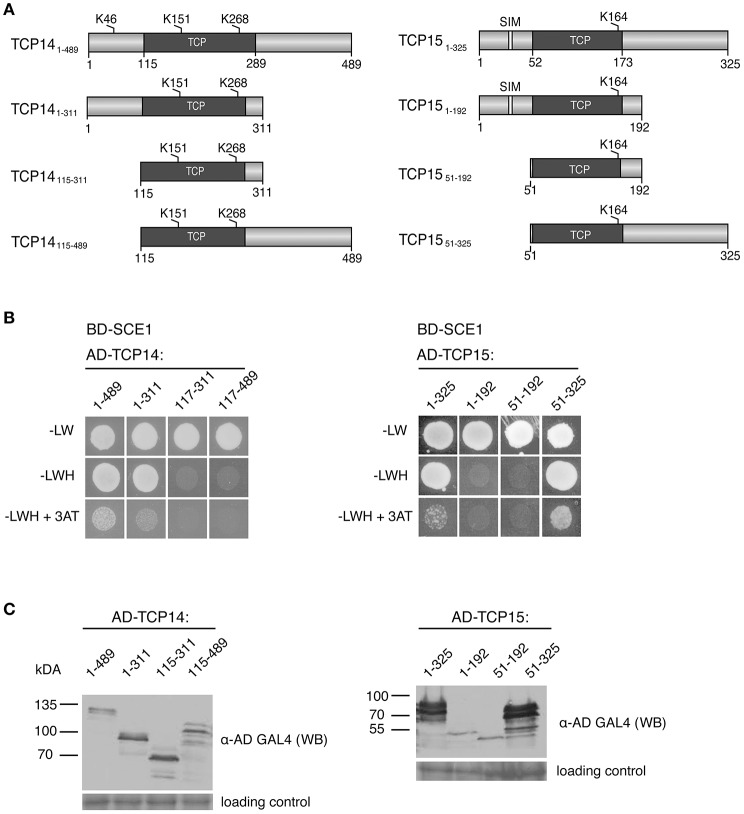
The DNA-binding domain of TCP14 and TCP15 is required but not sufficient for the interaction with SCE1 in the Y2H assay. **(A)** Schematic representation of TCP14 and TCP15 fragments used in the Y2H assay. The numbers denote the residue positions in the full-length protein. Gray boxes represent the DNA-binding domain of the TCPs, while the white boxes depict putative SIMs. The predicted SUMO acceptor sites are indicated by K and the residue position. **(B)** Y2H protein interaction test between BD-SCE1 and fragments of TCP14 and TCP15. Yeast growth was scored after 3 days at 30°C. **(C)** Immunoblot showing the expression of the AD-TCP14/15 fragments in yeast. The fragments were detected using an antibody against the GAL4 AD. The molecular weights (kDa) for the protein standards are indicated on the left. The membranes were stained with Ponceau S to confirm equal loading of the protein extracts.

As several TCPs interacted with SCE1, we wondered whether the TCP domain itself is sufficient or essential for the interaction with SCE1. First, we analyzed the TCP protein sequences to see if, in general, the TCP domains contain a shared/conserved SIM or SAS motif. The position of the TCP domains was based on the prediction by PFAM (http://pfam.xfam.org), while SIM and SAS motifs were again detected with GPS-SUMO (Table 2). This yielded no indication for the presence of a conserved SIM or SAS motif in TCP domains. We then designed three fragments for TCP14 and −15 and tested if they could still interact with SCE1 (Table 2, Figure 6A). In the case of TCP14 the N-terminal moiety including the TCP domain (TCP14_1−311_) was needed for the interaction with SCE1, while for TCP15 the C-terminal moiety (TCP15_52−325_) including the TCP domain was needed for the interaction with SCE1 (Figure 6B). In both cases, the TCP domain itself was not sufficient for the interaction. Also the predicted SIM in TCP15 (positions 29–33) appears not to be required for the interaction with SCE1, as the fragment TCP15_1−192_ failed to interact with SCE1. As a control, we confirmed that the different TCP14 and −15 fragments accumulate in yeast (Figure 6C). Combined, this suggests that the interaction between the TCP proteins and SCE1 is not an intrinsic property of the TCP domain, but rather involves recognition of SUMO acceptor sites (SAS) by SCE1, which are positioned outside the TCP domain in the TCP proteins studied here.

**Table 2:** Prediction of the SIM and SAS motifs for the here studied TCP family members.

**AGI code**	**Name**	**TCP domain^a^ (Position)**	**Predicted SIM^b^**		**Predicted SAS^b^**	
			**Position**	**Peptide^c^**	**Position**	**Peptide^c^**
At1g67260	TCP1	85–239	–	–	87 171 207	KEIKKVV**K**KDRHSKI DVEQEEE**K**EEDDNGD KAGIRKK**K**SELRNIS
At1g53230	TCP3	47–155	35–39	NGGGCG**EIVEV**QGGHIVRS	381	EEHGGDN**K**PSSASSD
At1g58100	TCP8	54–220	30–34	RQLVDAS**LSIVP**RSTPPED	382^**^	NAVEHQE**K**QQQSDHD
At3g47620	TCP14	115–289	–	–	K46^**^ 151^**^ 268^**^	FPFQLLG**K**HDPDDNH LTRELGH**K**SDGETIE LNFHNPT**K**QEGDQDS
At1g69690	TCP15	52–173	29–33	TSSSSTS**LAIIS**TTSEPNS	164	HQHQVRP**K**NESHSSS

a *The coordinates of the TCP domains were predicted using PFAM (EMBL-EBI)*.

b *SIM and SAS motifs were predicted using GPS-SUMO 2.0*.

c *In bold is indicated the hydrophobic residues that form the putative SIM or the lysine residue that acts as SUMO acceptor*.

** *Predicted with low threshold stringency*.

## Discussion

Here we identified 76 Arabidopsis TFs as putative substrates for SUMO conjugation and/or as interactors of the Arabidopsis SUMO machinery. Of these proteins 73 proteins are entirely novel SUMO substrates and/or interactors, while 32 proteins are novel interactors of SIZ1. Only two of these interactors were previously identified as SUMO substrates in Arabidopsis (Miller et al., 2010), namely ETHYLENE RESPONSE FACTOR 6 (ERF6) (AT4G17490) and the homeodomain transcription factor protein BEL1 (BELL1, AT5G41410). In our screen ERF6 and BEL1 interacted with SCE1 (Table 1), corroborating that they are direct substrates for SUMO conjugation. We found very little overlap between our set of Y2H interactors and the set of Y2H interactors reported by Elrouby et al. (Elrouby and Coupland, 2010; Elrouby et al., 2013). These authors reported for nine of the clones tested here that they interact with SCE1, ESD4, SUMO1, and/or SUMO2. Of these nine proteins we only found one to interact, namely STUbL6 (SUMO-targeted Ubiquitin ligase 6, AT2G44410), which interacted with SUMO1 and −2 (Elrouby et al., 2013). Elrouby et al. reported that STUbl6 interacted with a catalytically dead variant of ESD4 (ESD4^C448S^), but they did not report it as an interactor of SUMO1 or−2.

One explanation for not finding the same interactors in both screens could be the fact that we used the pDEST22/32 system, which provides a more stringent selection (due to lower protein levels of the prey proteins) (Rajagopala et al., 2009), while Elrouby et al. used the pGBKT7-pGADT7 system. Consequently, certain relevant interactions might potentially be lost in comparison to the pGBKT7/pGADT7 system. In agreement, we lost the interaction of BEL1, ERF6 and STUbL6 when we applied more stringent selection (1 mM 3-AT). These observations suggest that the interactions with SUMO and its machinery are relatively weak and that high protein levels might be important to pick up these interactions in the Y2H assay.

### TCP TFs as substrate for SUMOylation

Especially, the TCP TFs were enriched in our set of interactors (Figure 1). This protein family consists of 24 members in Arabidopsis, which is subdivided into two classes: Class I (also known as the PCF class) and Class II (further divided into the CYC/TB1 and CIN classes) (Martín-Trillo and Cubas, 2010). We found that 11 out of the 17 TCPs tested here interacted with our baits and that they belonged to both classes. We continued with the five strongest interactors (TCP1,−3,−8,−14, and −15), for which we demonstrated that (i) they interacted via the catalytic site of SCE1, (ii) they interacted with SCE1 in BiFC, and (iii) they were readily sumoylated in a reconstituted sumoylation assay in *E. coli*. TCP sumoylation was not class specific, as members of both classes were readily modified. Arguably more importantly, we found that several TCPs (at least TCP3,−8,−14, and −15) showed a redistribution into nuclear bodies and/or punctate structures when they interacted with SCE1 (rather than a diffuse signal in the nucleoplasm). Such nuclear structures were previously reported for several TCPs. For example, homodimerization of TCP8 triggers the formation of nuclear aggregates, which was linked to the presence of an intrinsically disordered region in its C-terminus (Valsecchi et al., 2013). Also TCP4 localizes to large irregular structures, while in a BiFC interaction with GIGANTEA (GI) it localizes in nuclear speckles (Kubota et al., 2017). In a similar manner, both TCP8 and TCP14 interact with SRFR1 in nuclear punctae in the BiFC assay, while the SRFR1/TCP15 pair shows a diffuse signal in the nucleoplasm (Kim et al., 2014). On the other hand, TCP8 did not form nuclear punctae in a BiFC assay with its interacting protein PNM1, a pentatricopeptide repeat (PPR) protein (Hammani et al., 2011). The first reports match with our observations that these three TCPs can localize to substructures in the nucleus, but clearly different (expression) conditions apparently affect the nuclear distribution of the TCPs.

In particular we found that TCP15 was less prone to localize to large nuclear bodies (NBs). Yang and coworkers demonstrated that TCP14 localizes to NBs and that this depends on its DNA-binding ability (Yang et al., 2017). Moreover, these TCP14 NBs are recruited to JAZ3-degradation bodies by the bacterial effector protein HopBB1, a type III bacterial effector protein of the plant pathogen *Pseudomonas syringae*. Future work should focus on what the consequence of SUMO conjugation is on the nuclear localization of these individual TCPs and the biological relevance of the formation of these TCP-containing nuclear punctae/bodies. In particular, transgenic lines are needed in which the various TCPs are expressed from their native promoters in null mutant backgrounds. That would allow studies on the sub-nuclear localization of the native TCP protein pool in the presence/absence of high SCE1 levels. This is very relevant to studies on the role of TCP sumoylation in e.g., plant development and immunity (reviewed by Danisman, 2016).

Using GPS-SUMO, we could predict SASs but not SIMs in TCP1 and TCP14, while the protein sequence of TCP3,−8, and −15 contained both SAS and SIM motifs. In order to map the SCE1-binding sites on TCP14 and −15, we expressed different fragments of TCP14 and −15. For TCP14, we found that the N-terminal part is needed for the interaction with SCE1, which includes the putative SUMO acceptor site (Lys46). We also attempted to map the SCE1 interaction site for TCP15 using three overlapping fragments. The C-terminal fragment of TCP15 appeared to be important for protein stability of TCP15, as deletion of this C-terminal part resulted in less protein accumulation in yeast. This reduced protein stability of both TCP15_1−192_ and TCP15_51−192_ hampered our attempts to map the SCE1 interaction site on TCP15. However, the fragment TCP15_51−325_ could still interact with SCE1. Interestingly, this fragment lacks a putative SIM, corroborating once more that TCP15 would be a direct substrate for SCE1-mediated SUMOylation via its catalytic pocket.

### Does TCP sumoylation affect SA and GA signaling?

In general, TCPs act as transcriptional regulators that can either induce or repress gene expression depending on their interactions with other proteins (Hervé et al., 2009). By now the TCPs have been shown to control cell proliferation, gametophyte development and embryogenesis, seed germination, jasmonic acid and salicylic acid synthesis, and photomorphogenesis (reviewed by Martín-Trillo and Cubas, 2010; Wang et al., 2015). TCPs are also important regulators of plant defense responses. For example TCP8/TCP9 regulate the expression of *ISOCHORISMATE SYNTHASE 1* (*ICS1*) (Wang et al., 2015), which encodes the key enzyme for salicylic acid biosynthesis in response to biotic stress. This link of TCP8/TCP9 with SA synthesis could be relevant as a T-DNA insertion knock-out mutant of the SUMO E3 ligase SIZ1 displays constitutive defense signaling due to high SA levels (Lee et al., 2007; van den Burg et al., 2010; Gou et al., 2017). In addition, TCP15 was reported to bind to the promoter of the immune receptor gene *SNC1* (*SUPRESSOR OF npr1-1, CONSTITUTIVE 1*) and modulate its expression (Zhang et al., 2017); Interestingly, this gene is important for the SIZ1 auto-immune defense phenotype (Gou et al., 2017).

Some of the known interactors of TCPs are also SUMO conjugation targets. For instance, the DELLA proteins, which interact with the Class I TCPs including TCP14 (Davière et al., 2014), are also SUMO substrates; DELLAs are conserved transcriptional repressors of gibberellin (GA) signaling, (Zentella et al., 2007; Conti et al., 2014). Binding of GA to the gibberellin receptor GID1 (GIBBERELLIN INSENSITIVE DWARF1) enhances the interaction between GID1 and DELLAs, resulting in rapid degradation of the DELLAs via the Ubiquitin-26S proteasome pathway. It was demonstrated that the interaction between DELLAs and GID1 is SUMO-dependent; the DELLAs are SUMO substrates, while GID1 contains a functional SIM (Conti et al., 2014; Nelis et al., 2015). DELLAs block the DNA-binding domain of Class I TCPs and thereby reduce their binding to their target promoters (Davière et al., 2014). Future studies should expose whether the interactions between DELLAs and TCPs are SUMO conjugation-sensitive. However, localization of the DELLA·TCP14 complex to any subnuclear structures was not detected (Davière et al., 2014), suggesting that the formation of this DELLA·TCP14 complex might be independent of SUMO (conjugation).

### TCP·Topless·EAR repressor complexes are decorated with SUMOs

The ethylene-responsive element binding factor (ERF)-associated amphiphilic repression (EAR) motif is a transcriptional repression motif found in members of the ERF, C2H2, and IAA families of transcriptional regulators (Ohta et al., 2001; Kagale et al., 2010). EAR-containing repressors interact physically with TOPLESS and TOPLESS-related proteins (TPL/TPRs), and together they affect chromatin modification via HDA19-dependent histone deacetylation (Long et al., 2006; Causier et al., 2012). Recently, TCPs were proposed to form ternary complexes with TIE1 (TCP INTERACTOR CONTAINING EAR MOTIF PROTEIN1) and TPL/TPRs to regulate leaf development (Tao et al., 2013). The nuclear transcription repressor TIE1 recruits the TPL/TPR co-repressors through its C-terminal EAR-motif, and in this manner represses the activities of TCPs that interact with the N-terminal domain of TIE1. Interestingly, many proteins that reside in the TPL/TPR corepressor complex appear to be sumoylated *in vivo* including TPL and its closest homologs (TPR1-4) (Miller et al., 2010; Mazur and van den Burg, 2012). Our finding that a substantial number of TCPs interacted with the SUMO machinery raises the possibility that SUMO controls the formation and/or activity of this TCP·TIE1·TPL complex. In support, we found that the TCPs only interacted with the catalytically active form of SCE1 and that this complex was localized to nuclear foci. In fact, overexpression of SCE1 is already sufficient to translocate the five tested TCPs to nuclear foci, meaning that enhanced SUMO conjugation activity may induce formation of TCP nuclear foci. Importantly, the TIE1·TCP10 complex also localizes to nuclear foci (Tao et al., 2013), which resemble the TCP-SCE1 foci seen here. The exact mechanism how SUMO affects localization and the biological function of these protein complexes will, however, require additional research.

### Sumoylation regulates a broad range of cellular processes

Finally, two interesting interactors of SIZ1 are ELONGATED HYPOCOTYL 5 (HY5) and a homolog of HY5 (HYH). Both belong to the basic leucine zipper (bZIP) TF family and they function in light/temperature-regulated and abscisic acid (ABA)-regulated transcriptional activation. HY5 is targeted for degradation by the ubiquitin E3 ligase COP1 in the dark (Ang et al., 1998) and regulates the expression of the gene *ABA-INSENSITIVE 5* (*ABI5*) by binding to its promoter (Chen et al., 2008). Interestingly, ABI5 and COP1 were recently shown to be sumoylated in a SIZ1-dependend manner (Miura et al., 2009; Lin et al., 2016). ABI5 and HY5 also localize to nuclei. However, when co-expressed with COP1 they are redirected to NBs. Whether COP1, ABI5 and HY5 co-localize to NBs to form a functional complex in a SIZ1-depended manner is the subject of future studies. It would be interesting to investigate whether SIZ1-dependent sumoylation of COP1 also affects HY5 localization.

We also identified several NAC and AP2-ERF family members. NACs are implicated in controlling plant development and responses to biotic and abiotic stresses (Nakashima et al., 2012; Nuruzzaman et al., 2013). For example, NAC020, NAC048, and NAC088 are involved in multicellular organ development, while NAC13 regulates the oxidative stress response. AP2-ERFs regulate signaling in response to the plant hormones ethylene and brassinosteroids (Alonso et al., 2003; Hu et al., 2004), and the response to biotic and abiotic stresses (Li et al., 2011; Mizoi et al., 2012). To conclude, our study further extends the range of plant processes affected by sumoylation.

## Materials and methods

### Construction of yeast two-hybrid vectors

All molecular techniques were performed using standard protocols (Sambrook and Russell, 2001); the primers used in this study are listed in the Table S1. The cDNA clones of *SUMO1* (AT4G26840) (encoding for the mature SUMO; residues 1–93), *SUMO2* (AT5G55160.1) (mature SUMO; residues 1–93), *SUMO3* (AT5G55170) (mature SUMO; residues 1–93), *Ulp1C* (AT1G10570), *ESD4* (AT4G15880), and *SIZ1* (AT5G60410) were amplified from previously published plasmids. Wild type *SCE1* (AT3G57870) was recombined in the Y2H plasmids from pENTR/SD-dTOPO clone U15589 (Arabidopsis Biological Resource Center). Primers containing the *attB1* and *attB2* Gateway recombination sites were used to amplify the various gene sequences (see Table S1). Truncated SUMO proteins, SUMO1^Δ*GG*^, SUMO2^Δ*GG*^, and SUMO3^Δ*GG*^ (residues 1–91 for all 3 SUMOs), were obtained by PCR-amplification of *SUMO1, SUMO2*, and *SUMO3* gene sequences using the Phusion DNA polymerase (Thermo Fisher) with the primer pairs: FP3393/FP3962, FP3379/FP3381, and FP3394/FP3392, respectively. The truncated *ULP1C* (1–345), *ESD4* (1–285), and *SIZ1*(1–536) were obtained by PCR-amplification using the primers: FP5011/FP5700, FP3219/FP5692, and FP3407/FP5250, respectively. Clones with point mutations in *ULP1C* (C512S) and *ESD4* (C448S) were generated using site-directed mutagenesis with the primer pairs: FP5699/FP5698 and FP5691/FP5690, respectively, following the QuikChange protocol (Agilent technologies). The *SCE1 C94S* clone was provided by Nam Hai Chua (Rockefeller University, New York) (Lois et al., 2003). The cDNA clones of *SCE1 R14E/R18E/H20D, SCE1 Y87A/S89A/C94S/D129A*, and *SCE1 P70A/P106A* were synthesized by Eurofins Genomics. The truncations of TCP14 and TCP15 were obtained with the appropriate primer pairs (Table S1). The resulting PCR products were recombined with the Gateway vector pDONR221 (Thermo Fisher) using BP Clonase II (Thermo Fisher) and checked by sequencing. All the cDNA clones were introduced in the pDEST32 (Thermo Fisher) vector to obtain GAL4 BD-fusion constructs by Gateway LR Clonase II reaction (Thermo Fisher) and the final clones were verified by DNA sequencing. All primers (Table S1) were synthesized by Eurofins.

### Construction of binary vectors for agro-infiltrations

The gene constructs used for the Y2H were subjected to the BP reaction to move them to the Gateway vector pDONR207 (Clontech) using BP Clonase II enzyme. For *in planta* protein localizations, the different cDNA clones were introduced in the Gateway binary pGWB452 (N-terminal GFP tag) (Nakagawa et al., 2007). For the BiFC studies, the different cDNA clones were introduced into a pair of Gateway destination vectors: pSCYNE(R) (N-terminal half of S(CFP)3A consisting of the residues 1–173, referred as CFP^N^) and pSCYCE(R) (C-terminal half of S(CFP)3A consisting of the residues 156–239, referred as CFP^C^) in order to reconstitute the super Cyan Fluorescent Protein SCFP (Gehl et al., 2009). All LR reactions were performed using LR clonase II (Thermo Fisher) and the resulting clones were confirmed by sequencing.

### Construction of vectors for *E. coli* sumoylation assays

pACYCDuet:*SAE1/SAE2*, pCDFDuet:*SUMO1-SCE1* and pET28a:*MYB30* were obtained from Katsunori Tanaka (Okada et al., 2009). The *HsfB2b* cDNA clone was obtained from ABRC. *HsfB2b, TCP1, TCP3, TCP8, TCP14*, and *TCP15* present in pDONR207 were cloned into pET28a-DEST (an in house made derivative of pET28a) by LR reaction. All LR reactions were performed using LR clonase II (Thermo Fisher) and the resulting clones were confirmed by sequencing.

### GAL4 yeast two-hybrid protein-protein interaction assays

The yeast two-hybrid protocol as described by de Folter and Immink (2011) was followed. The pDEST32 clones were transformed into the *Saccharomyces cerevisiae* yeast strain PJ69-4α (James et al., 1996) using the standard lithium acetate (LiAc)/single-stranded carrier DNA (ssDNA)/polyethylene glycol 3350 protocol (Gietz and Woods, 2002). From each yeast transformation, three independent colonies were picked and grown on selective minimal medium (MM) supplemented with amino acid dropout solution lacking L-leucine (MM-L). The possibility of the BD-fusion clones to auto-activate the *GAL4* promoter was excluded by growing pDEST32 transformants on MM lacking L-leucine and L-histidine (MM-LH). The arrayed yeast library REGIA (Paz-Ares, 2002) was grown on MM lacking L-tryptophan (MM-W). The AD clones in the yeast library were expressed from pDEST22 (Thermo Fisher) destination vector. For yeast mating, both the BD (grown on liquid MM-L overnight) and AD clones were co-spotted with a 96-pin replicator on MM agar plates supplemented with full amino acid solution (MM). After 2 days the obtained colonies were resuspended in sterile water and transferred on double selective medium (MM-LW) to select for mated yeast. Interactions were scored on MM-LWH agar plates. Plates were incubated at 30°C for 3 days before being scored for protein-protein interactions. The yeast that had grown on MM-LWH was re-grown on MM-LWH and MM-LWH supplemented with 1 mM 3-Amino-1,2,4-triazole (3-AT). In order to confirm the identity of the AD insert, plasmid DNA was (re-)isolated from positive yeast colonies and sent for DNA sequencing.

### Protein isolation from yeast and detection using immunoblotting

Total protein was extracted from yeast according to the Yeast Protocols Handbook (Clontech; Yeast Protocols Handbook: http://www.clontech.com/xxclt_ibcGetAttachment.jsp?cItemId=17602). In brief, yeast cultures were grown in minimal medium with the appropriate amino acids supplemented. At OD_600_ = 0.6, the cells were collected and frozen in liquid nitrogen. One hundred microliters cracking buffer (8 M Urea, 5% w/v SDS, 40 mM Tris-HCl pH 6.8, 0.1 mM EDTA, 0.4 mg/ml bromophenol blue, with freshly added 1% β-mercaptoethanol, 1 × protease inhibitor cocktail [Roche], 1 mM PMSF) was added per 7.5 OD_600_ of frozen cell pellet together with glass beads to the frozen cells. The cells were then heated at 70°C for 10 min, vortexed for 1 min and then centrifuged for 5 min at 13,000 g. The resulting supernatant was boiled for another 5 min and loaded on a 10% SDS-PAGE gel. The separated proteins were transferred onto PVDF membranes (Immobilon-P, Millipore) using semi-dry blotting. Skimmed milk powder (5%) in phosphate-buffered saline (137 mM NaCl; 2.7 mM KCl, 10 mM Na_2_HPO_4_, 1.8 mM KH_2_PO_4_) supplemented with 0.1% Tween 20 (PBS-T) was used to block the membranes. Monoclonal antibodies against the GAL4 binding and activation domains (Clontech 630403 and 630402, respectively) were used at a dilution of 1:500 to detect the fusion proteins. The secondary antibody goat anti-mouse IgG conjugated with Horseradish peroxidase (Pierce 31430) was used at a dilution of 1:10,000. Protein bands were visualized using enhanced chemiluminescence (10 mL homemade ECL solution containing 100 mM Tris HCl pH 8.5, freshly supplemented with 50 μL of 250 mM Luminol in DMSO, 22 μL of 90 mM coumaric acid in DMSO, and 3 μL 30% H_2_O_2_ solution) detection on MXBE Kodak films (Carestream). Equal protein loading for the samples was confirmed by staining the blot with the dye Coomassie blue.

### TF family search and statistics

The grouping of the TF families was done using the Database of Arabidopsis Transcription Factors (http://datf.cbi.pku.edu.cn). The TCP domains were annotated using Pfam (EMBL-EBI). For the statistical analyses (TF enrichment test), a binomial test was used, where the expected value (probability of occurrence) is the number of TFs of a certain TF family present in the REGIA library divided by the total number of TFs in the REGIA library. The difference was considered to be significant when the *p*-value < 0.05.

### SUMO acceptor site (SAS) and SUMO interaction motif (SIM) enrichment analyses

For the prediction of SAS and SIM, we used the online tool GPS-SUMO 2.0 (http://sumosp.biocuckoo.org) with a medium stringency of 90% (Ren et al., 2009; Zhao et al., 2014). SAS were only predicted for SCE1 interactors, while SIMs were predicted for all the interacting baits identified. As control group, protein sequences of the total Arabidopsis proteome were downloaded from TAIR (https://www.arabidopsis.org). For statistical analyses, a two-category binomial test was used. The difference was considered significant at a *p*-value < 0.05.

### Agrobacterium mediated transformation assay and confocal microscopy

The *Agrobacterium tumefaciens* strain GV3101(pMP90) (Koncz and Schell, 1986) was transformed with the desired binary constructs by electroporation. Single colonies were grown until OD_600_ = 0.5 in low salt LB medium (1% Tryptone, 0.5% yeast extract, 0.25% NaCl, pH 7.0) supplemented with 20 μM acetosyringone and 10 mM MES (pH 5.6). Cells were pelleted and resuspended in infiltration medium (standard 1 × Murashige and Skoog nutrient medium with 10 mM MES pH 5.6, 2% w/v sucrose, and 200 μM acetosyringone). The cells were infiltrated into 4–5 week-old *N. benthamiana* leaves at an OD_600_ = 1.0. To suppress gene silencing, we co-infiltrated an *A. tumefaciens* strain GV3101 (OD_600_ = 0.5) that carried a binary plasmid (pBIN61; a pBIN19 derivative) to express the P19 silencing suppressor of the Tomato busy shunt virus (TBSV), (Voinnet et al., 2003). Protein expression was examined 2–3 days post-infiltration.

Accumulation of the GFP-tagged proteins and reconstitution of SCFP in *N. benthamiana* leaf epidermal cells was detected using a confocal laser-scanning microscope (Zeiss LSM510). All images were taken with a C-Apochromat 40x1.2 W Korr objective. GFP and RFP were excited with the 488 nm laser line of the Argon laser and the 543 nm line of the Helium-Neon laser, respectively. Subsequently, for GFP imaging, fluorescence was separated from excitation light by a dual dichroic filter, reflecting both 405 and 488 nm laser light. Two secondary dichroic filters were installed in the beam path, LP490 and LP570 nm, separating the emission light in different channels. Light reflected by the LP570 filter passed a 520–555 nm band pass filter before detection. For RFP imaging, fluorescence was separated from excitation light by a dual dichroic filter, reflecting both 488 and 543 nm laser light. The emission light was separated by a 635LP filter and the reflected light passed a 585–615 nm band pass filter before detection. The BiFC experiments were performed according to Gehl et al. (2009).

### *E. coli* sumoylation assay

The procedure was previously described by Okada et al. (2009). Briefly, the *E. coli* strain BL21(DE3) harboring the plasmid pACYCDuet:SAE1/SAE2 was co-transformed with pCDFDuet:SUMO1/SCE1 and a desired target protein expressed from pET28a-DEST (in which the Gateway CmR-ccdB cassette was inserted in the HindIII/XhoI sites giving an in-frame fusion with the T7-tag after recombination). To this end, the Gateway CmR-ccdB cassette was amplified with the primers FP3709/FP3710 and the amplicon was digested with HindIII/XhoI. To induce protein expression in the *E. coli* cells 0.2 mM IPTG was added to the cultures and the cultures were incubated for an extra 3 h at 22°C with 220 rpm agitation. Five hundred microliters of the cultures was spun down, boiled for 10 min in Laemmli sample buffer and loaded on a 12% SDS-PAGE gel. The separated proteins were blotted onto PVDF membranes (Immobilon-P, Millipore) using semi-dry blotting. Skimmed milk powder (5% w/v) in PBS-T was used as blocking agent for the membranes. Monoclonal antibodies directed against T7 (Novagen, 69522) were used at a dilution of 1:1,000. The secondary antibody goat anti-mouse IgG conjugated with Horseradish peroxidase (ThermoFisher 31430) was used at a dilution of 1:10,000. The proteins were visualized using enhanced chemiluminescence (ECL) and detected on MXBE Kodak films (Carestream).

## Author contributions

MM, AD, MvdG, BS, GV, and BB conducted the experiments. MM conducted the data analysis. HvdB and WG supervised the project with HvdB coordinating the research work. MM and HvdB drafted the paper and MM and BS produced the graphical outputs. All authors approved the final version of the manuscript.

### Conflict of interest statement

The authors declare that the research was conducted in the absence of any commercial or financial relationships that could be construed as a potential conflict of interest.
