# Supplementary material for: Arabidopsis TCP Transcription Factors Interact with the SUMO Conjugating Machinery in Nuclear Foci
Source: Front Plant Sci. 2017 Nov 30;8:2043. doi: 10.3389/fpls.2017.02043 (PMC5714883; doi:10.3389/fpls.2017.02043)
Supplement: Supplementary file 2 [file Presentation1.PPTX]

## Slide 1
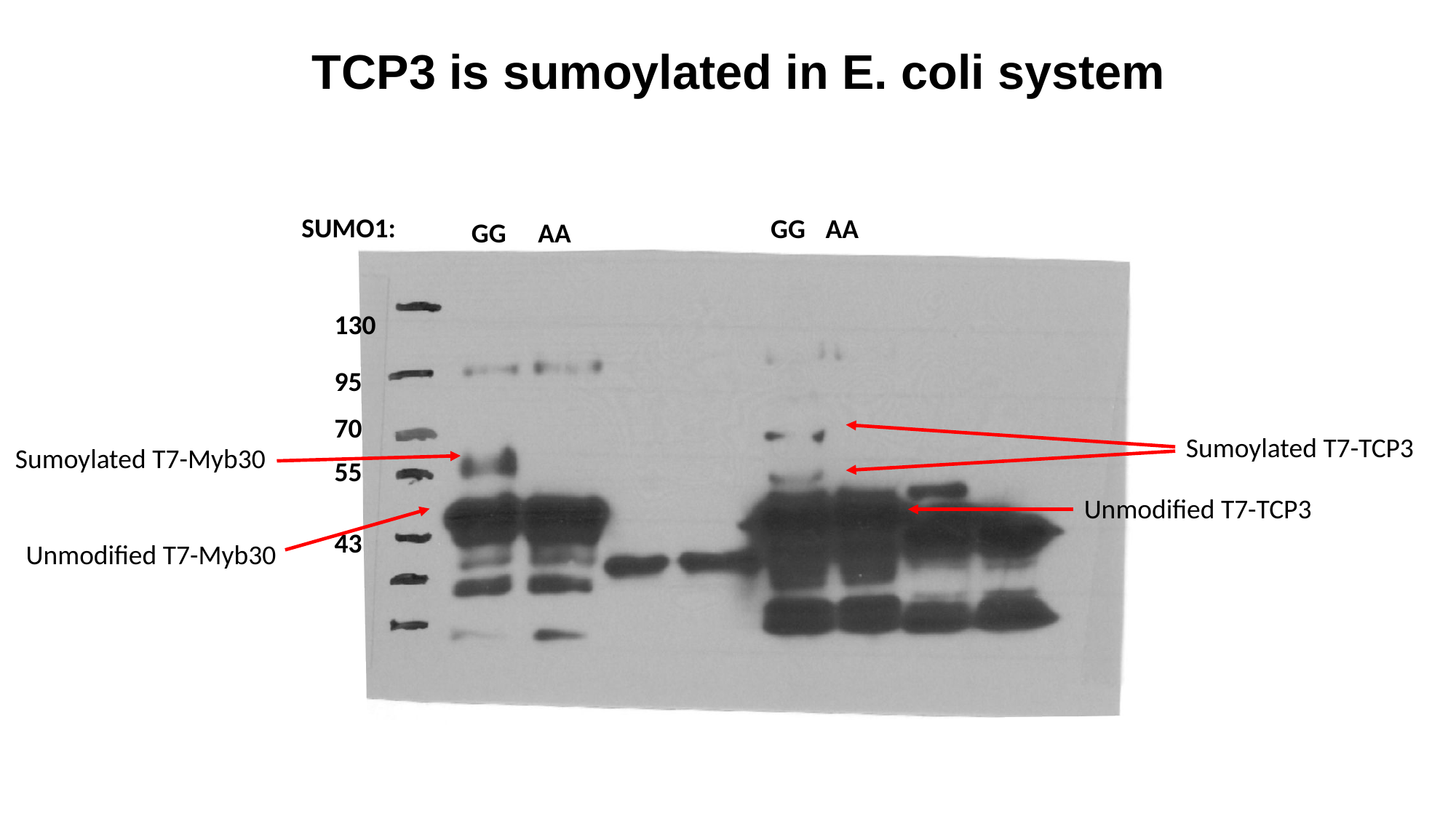

TCP3 is sumoylated in E. coli system
SUMO1:
GG
AA
GG
AA
130
95
70
Sumoylated T7-TCP3
Sumoylated T7-Myb30
55
Unmodified T7-TCP3
43
Unmodified T7-Myb30

## Slide 2
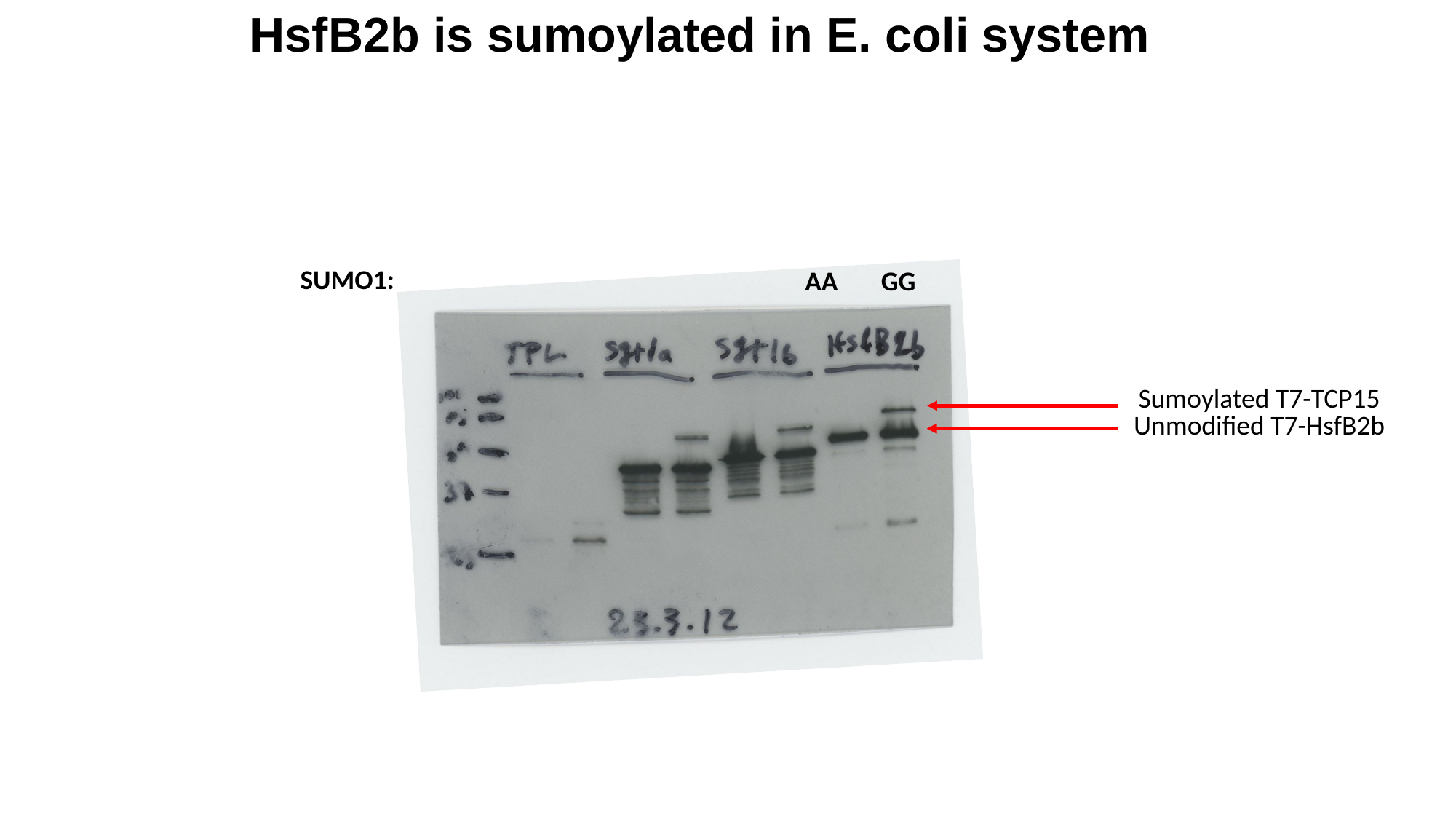

HsfB2b is sumoylated in E. coli system
SUMO1:
AA
GG
Sumoylated T7-TCP15
Unmodified T7-HsfB2b

## Slide 3
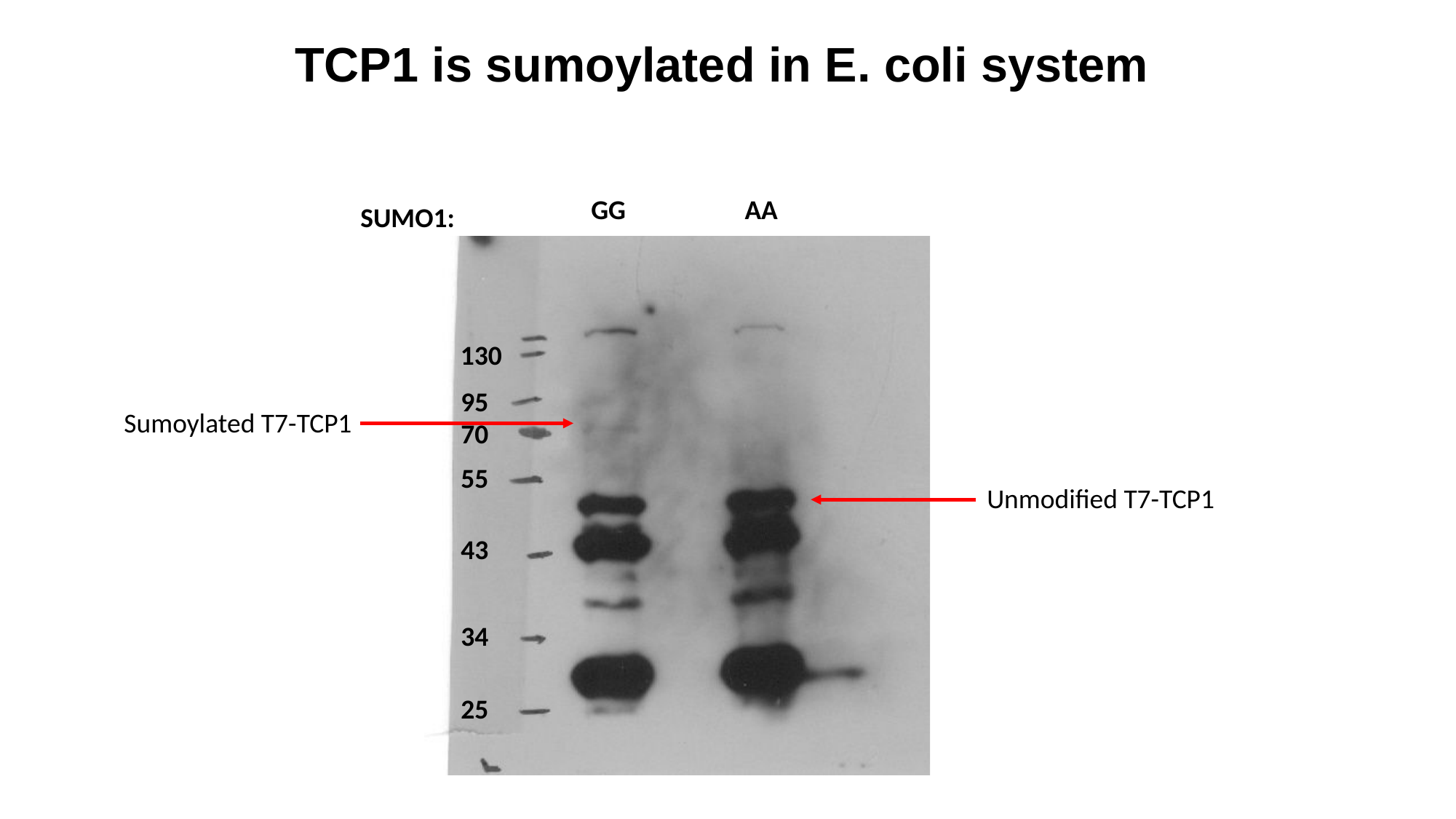

TCP1 is sumoylated in E. coli system
GG
AA
SUMO1:
130
95
Sumoylated T7-TCP1
70
55
Unmodified T7-TCP1
43
34
25

## Slide 4
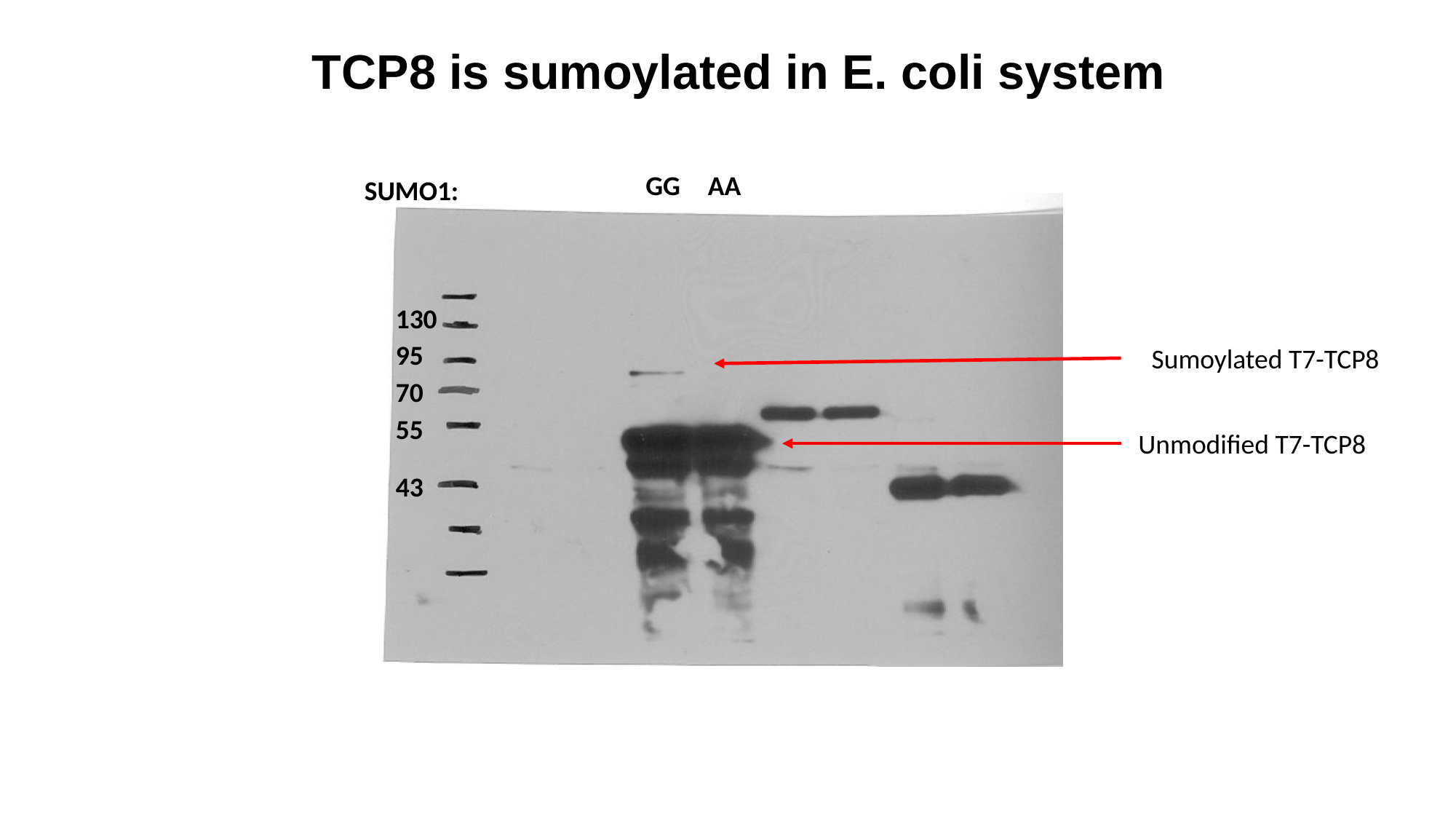

TCP8 is sumoylated in E. coli system
GG
AA
SUMO1:
130
95
Sumoylated T7-TCP8
70
55
Unmodified T7-TCP8
43

## Slide 5
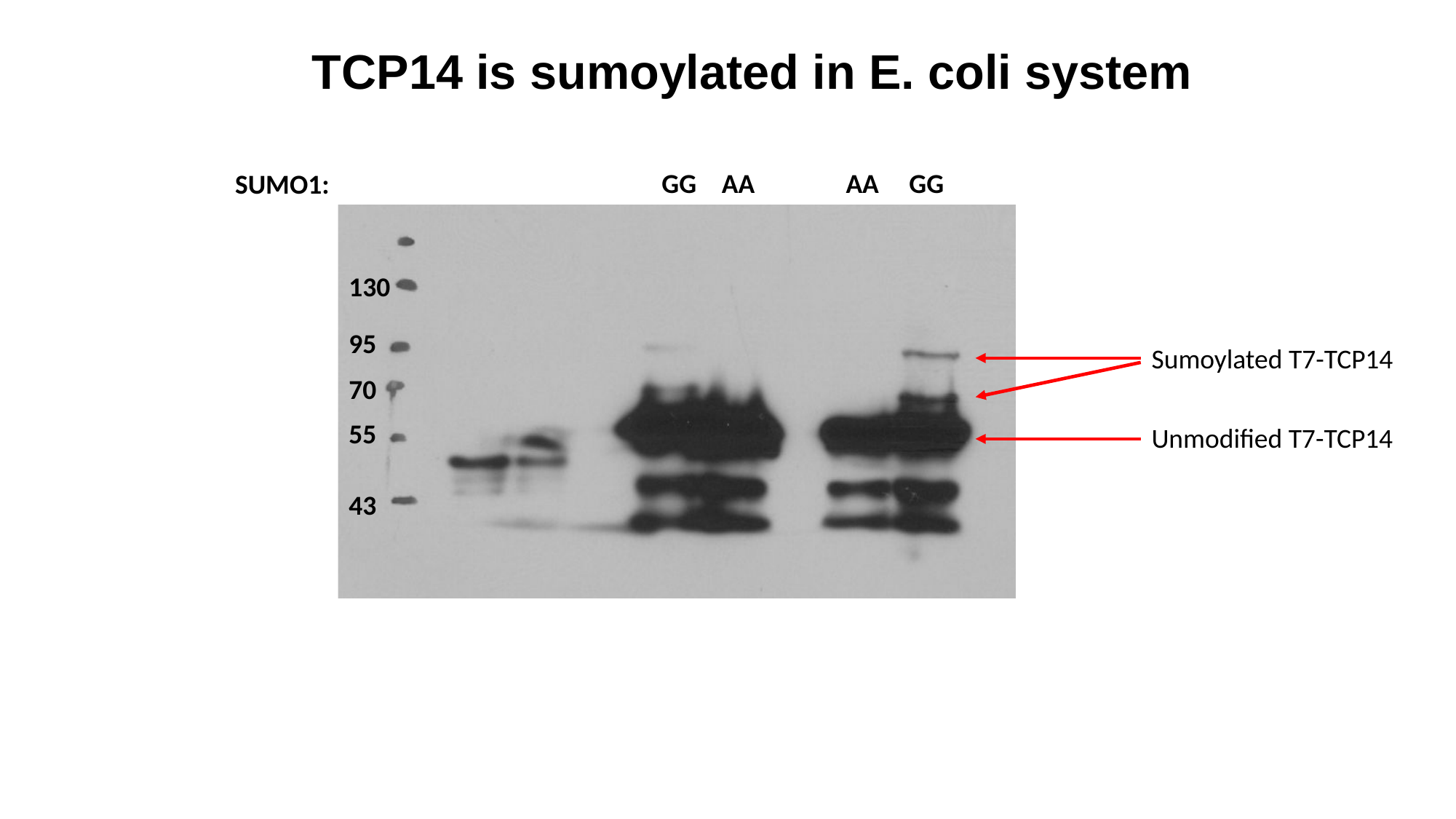

TCP14 is sumoylated in E. coli system
GG
AA
AA
GG
SUMO1:
130
95
Sumoylated T7-TCP14
70
55
Unmodified T7-TCP14
43

## Slide 6
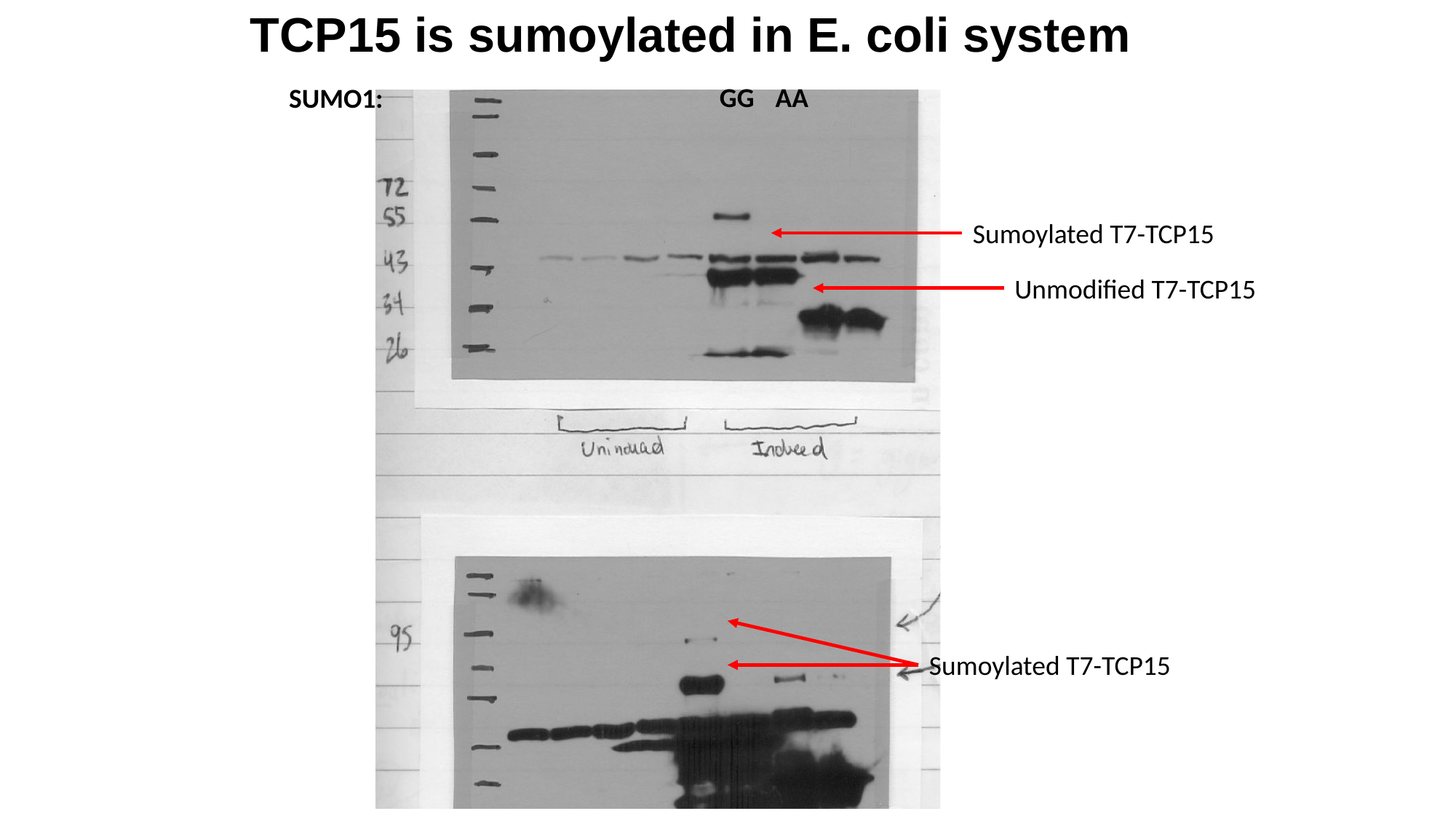

TCP15 is sumoylated in E. coli system
GG
AA
SUMO1:
Sumoylated T7-TCP15
Unmodified T7-TCP15
Sumoylated T7-TCP15
