# Supplementary figures and images for: Arabidopsis TCP Transcription Factors Interact with the SUMO Conjugating Machinery in Nuclear Foci
Source: Front Plant Sci. 2017 Nov 30;8:2043. doi: 10.3389/fpls.2017.02043 (PMC5714883; doi:10.3389/fpls.2017.02043)

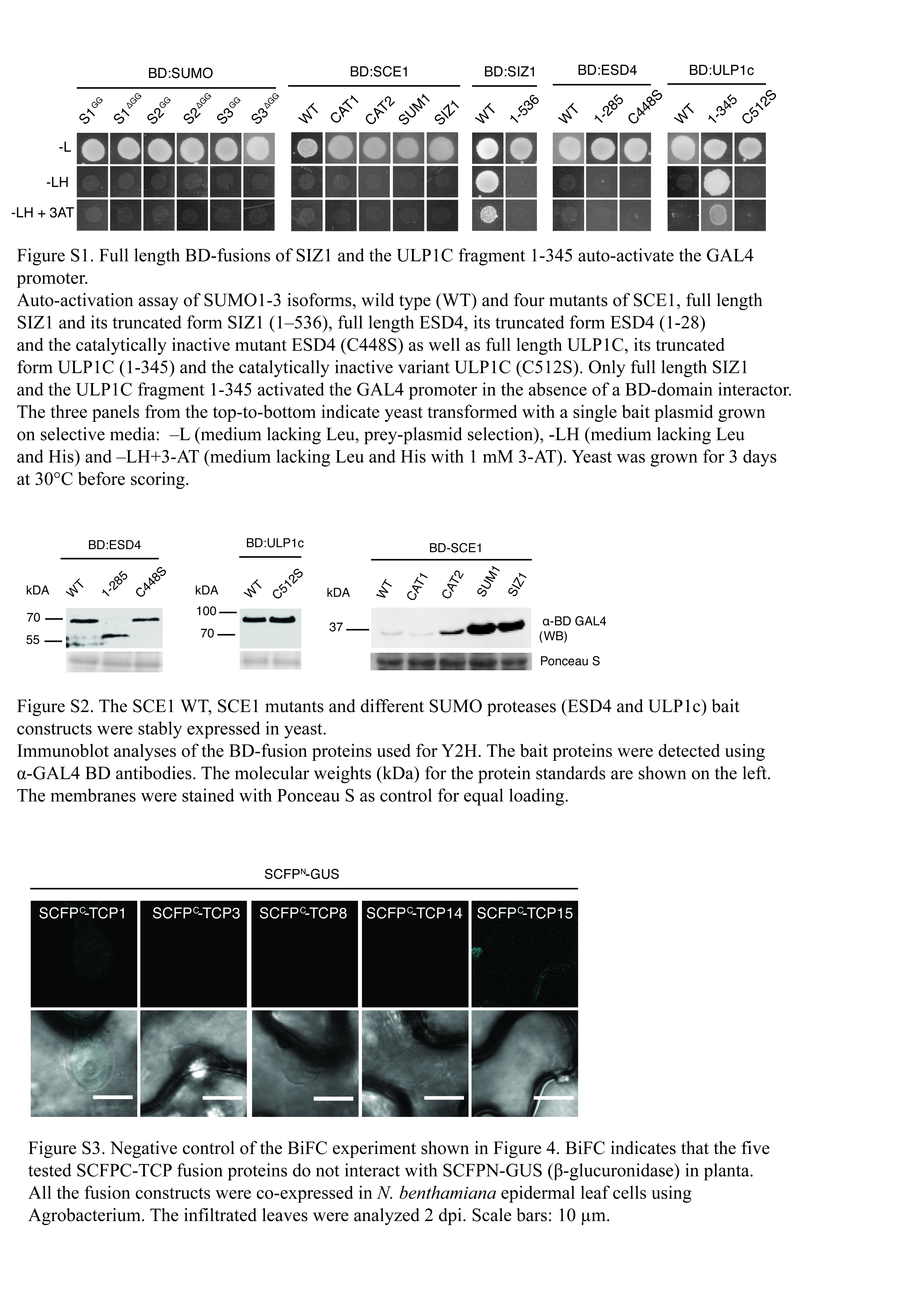

Supplement: Supplementary file 3 [file Image1.JPEG]
